# Improving the Skin‐Conformability of Wearable Continuous Glucose Monitors With Synthetic Hydrogel Electrodes

**DOI:** 10.1002/advs.202517501

**Published:** 2026-01-22

**Authors:** Binbin Cui, Shilei Dai, Ivo Pang, Dingyao Liu, Xinran Zhang, Jing Bai, Xinyu Tian, Shiming Zhang

**Affiliations:** ^1^ Department of Electrical and Electronic Engineering The University of Hong Kong Hong Kong SAR China; ^2^ State Key Laboratory of Pharmaceutical Biotechnology The University of Hong Kong Hong Kong SAR China

**Keywords:** continuous glucose monitor, enzyme membrane, hydrogel electrode, synthetic bioelectronics, wearable health

## Abstract

Synthetic bioelectronics is rapidly advancing, propelled by breakthroughs in synthetic biology and bioelectronics. This convergence is key to next‐generation wearable and implantable devices, enabling seamless integration with living systems. Here, we introduce an enzymatic hydrogel electrode (GelZymes) developed via a synthetic bioelectronic strategy to overcome the mechanical and interfacial limitations of conventional enzyme electrodes. GelZymes deliver two core advances: i) a monolithic and scalable 3D architecture that unifies the enzyme membrane and electrode, simplifying fabrication and eliminating interfacial instability; and ii) tissue‐like viscoelasticity—combining stretchability and adhesiveness—rarely achievable with rigid enzyme membranes. GelZymes are synthesized through three steps: engineering a stretchable, mixed‐conducting 3D hydrogel; implementing an enzyme‐compatible, cascading crosslinking scheme to immobilize enzymes within the network; and balancing the trade‐off between electronic/ionic conductivity and the density of redox‐active enzyme sites to maximize bio‐electrochemical performance. We further show that GelZymes enable a shift from invasive, tissue‐interfaced biosensing to noninvasive, tissue‐integrated biosensing, offering a practical pathway to bridge current biosensor technologies with living systems.

## Graphical abstract

A synthetic hydrogel electrode for skin‐conformable enzymatic biosensing is presented, featuring a stretchable, tape‐shaped continuous glucose monitor for non‐invasive diabetes management.

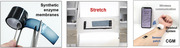

## 1 Introduction

Biosensors, initiated by Leland Clark's invention of the first glucose biosensor in the 1950s [1], are key bio‐analytical tools that convert biological reactions into quantifiable signals [2]. A biosensor comprises three core components: a bioreceptor, a transducer unit, and a signal processing system. In glucose biosensors, the bioreceptor is an enzyme membrane that catalyzes glucose oxidation on the electrodes, converting it into detectable electronic signals [3, 4]. In the past decades, different generations of glucose enzyme membranes have been developed, including oxygen‐based enzyme membranes (first generation) [5], mediator‐based enzyme membranes (second generation) [6], and direct electron transfer (DET) membranes (third generation) [7]. These membranes show high sensitivity and selectivity and have been widely used in making glucose sensors for research and applications.

Despite these advances, these enzyme membranes, which are prepared by immobilizing enzymes with plastic agents [8], are mechanically rigid and fragile, causing mechanical mismatches when interfacing with soft living systems. Therefore, the development of stretchable, or more advanced viscoelastic, enzyme membranes that enable seamless integration with soft biological elements has been a focus of research in recent years [9, 10, 11, 12, 13]. However, such enzyme electrodes remain rarely reported, primarily due to the lack of suitable material solutions.

In recent years, stretchable hydrogels based on 3D multi‐network synthesis strategies have drawn significant attention for their applications in soft bioelectronics [14, 15]. They have been widely used as electrodes for biophysical or bioelectrical sensing applications [16, 17, 18]. However, little research has been dedicated to exploring their capabilities for biochemical sensing [9]. Particularly, endowing those 3D, viscoelastic hydrogels with glucose‐sensing abilities could pave the way for developing next‐generation continuous glucose monitors (CGMs) in new forms of patches or textiles. Such 3D, stretchable, and enzymatic hydrogel membrane (GelZyme) would promote a paradigm shift from minimally invasive, needle‐based CGMs to non‐invasive, skin‐attached CGMs.

Despite promising, the development of GelZymes faces significant material challenges, particularly in preserving enzyme activity during complex hydrogel synthesis processes. Enzymes are sensitive to both chemical reactions and processing conditions, such as temperature and pH values [19]. Additionally, achieving a functional GelZyme requires balancing multiple inherently conflicting properties, including electrical conductivity, glucose diffusion properties, enzymatic activity, as well as mechanical stretchability.

**FIGURE 1:**
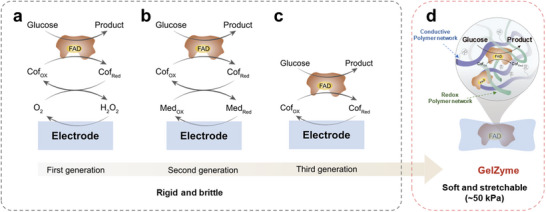
GelZymes address the mechanical mismatch between existing enzyme electrodes and living systems. A comparison between current glucose biosensing enzyme electrodes and the proposed GelZymes. a) First‐generation glucose sensors rely on O_2_‐mediated electron transfer between the redox center, flavin adenine dinucleotide (FAD), of the glucose oxidase enzyme and the electrodes. b) Second‐generation biosensors use artificial mediators to facilitate electron transfer. c) Third‐generation biosensors aim for direct electron transfer by minimizing the distance between the FAD and the electrode. d) Stretchable, soft GelZymes, for the first time, integrate the enzyme membrane and the electrode into a single 3D structure, simplifying fabrication, eliminating interface stability issues, and overcoming mechanical limitations through the use of multinetwork hydrogel designs.

Here, we report the successful development of GelZymes, which are viscoelastic, low‐modulus and stretchable, addressing the mechanical mismatch between enzyme electrodes and living systems (Figure 1). They are synthesized through the following key processes: i) synthesizing a 3D, multi‐network hydrogel as an enzyme‐loading framework, with one network for stretchability and another for electron conduction; ii) grafting glucose oxidase enzymes onto the hydrogel framework by developing an enzyme‐compatible processing workflow; and iii) grafting mediators onto the hydrogel network to promote electron transfer efficiency between the enzyme's active site, flavin adenine dinucleotide (FAD), and the electron‐conducting networks within the hydrogel.

The GelZymes exhibits effective glucose‐sensing capabilities. Their unique viscoelasticity, derived from the hydrogel, enables it to be shaped into various freestanding forms, such as fibers and patches, with high elasticity and robustness—qualities essential for tissue‐integrated applications. As a proof of concept, we demonstrated Tape‐CGM, a non‐invasive glucose monitoring system for sweat, by simply sticking the GelZyme on a commercially available medical tape. This approach highlights GelZymes’ unique potential to advance biosensing from invasive, interfaced systems to non‐invasive, integrated solutions.

## 2 Results and Discussions

### 2.1 Design Strategy of GelZymes

Although different generations of enzyme membranes have been proposed based on their distinct electron transport mechanisms, all such membranes remain fragile and lack elastoadhesiveness [1, 2, 5, 6, 20, 21]—properties essential for mitigating motion artifacts in skin‐interfaced biosensing applications.

Below are two main reasons for these limitations: i) Enzyme species have to be blended into non‐stretchable, rigid polymers to ensure efficient charge transfer [6]; and ii) Enzyme membranes and electrodes are assembled using a layer‐by‐layer architecture [5], which causes interface stability issues under motion‐induced body deformations.

**FIGURE 2:**
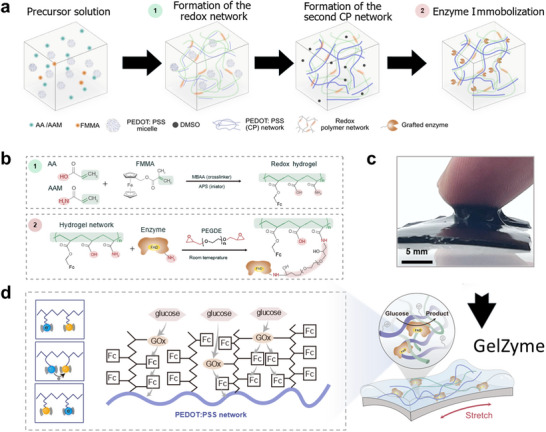
Process flow of the synthesis of the GelZyme. a) The components needed to assemble the stretchable PAA/PAAM network and the conductive PEDOT:PSS network are blended together. The stretchable PAA/PAAM hydrogel is formed via photo‐crosslinking, with the functionalized ferrocene mediator crosslinked into the network (1). The sample is then immersed in a high‐binding‐point organic solvent, such as DMSO, which facilitates the formation of the secondary conductive PEDOT:PSS network. Finally, functionalized enzymes, which are crosslinkable to the PAAM, are incorporated into the 3D network through a chemically and enzyme‐friendly immersion process at room temperature (2). b) Chemistry of GelZyme synthesis: stretchable redox hydrogel synthesis and enzyme immobilization. c) Real picture of the GelZyme, demonstrating its high viscoelasticity. d) Enzymatic reaction process of the GelZymes. Glucose molecules are first catalyzed by glucose oxidase, transferring electrons to the ferrocene groups embedded in the PAAM network. The mediator groups facilitate electron transfer through collision, and the PEDOT:PSS conducting network ultimately receives the electrons, finalizing the glucose sensing process.

We developed a strategy to synthesize the GelZyme that can simultaneously address these inherently conflicting challenges, as detailed below: i) First, we assembled an elastoadhesive and conductive hydrogel electrode using a multi‐network approach (Figure 2a); ii) Instead of a separate enzyme layer, we crosslinked the enzyme species directly into the same hydrogel electrode, thereby eliminating the interfacing issues between the membrane and the electrodes (Figure 2b); and iii) We maximized enzyme efficiency by promoting charge transport between the enzyme sites and the conductive hydrogel, achieved by controlling the concentrations of individual components and the processing conditions.

### 2.2 Synthesis of the GelZyme

The synthetic bio‐electrochemical processes of the GelZyme, following the above strategies, are illustrated in Figure 2a. They are composed of the following elements: i) A stretchable hydrogel network based on acrylic acid (AA)/acrylamide (AAM) precursors; ii) A conductive hydrogel network employing poly(3,4‐ethylenedioxythiophene) polystyrene sulfonate (PEDOT:PSS) conductive polymers; and iii) Functionalized enzyme species and ferrocene mediators, crosslinkable to the stretchable hydrogel network.

The detailed procedure to synthesize the GelZyme is as follows: First, the components required to assemble the stretchable poly(acrylic acid) (PAA)/polyacrylamide (PAAM) network and the conductive PEDOT:PSS network are blended together. The formation of the stretchable PAA/PAAM hydrogel, polymerized using an initiator [22], was verified by Fourier‐transform infrared spectroscopy (FTIR). The characteristic peaks in the spectra of the PEDOT:PSS thin film and GelZyme films confirm the successful incorporation of enzyme (Figure S1a). Simultaneously, the mediator, functionalized ferrocene, is also crosslinked into the stretchable network. Second, the sample is immersed in a high‐binding‐point organic solvent dimethylsulfoxide (DMSO), which facilitates the formation of the secondary conductive PEDOT:PSS network [23, 24]. The final step involved the incorporation of functionalized enzymes into the 3D network, where they were crosslinked to the PAA/PAAM matrix. This was achieved through a chemically mild, enzyme‐compatible immersion process conducted at room temperature (Figure 2b,c). Evidence for successful enzyme immobilization was also obtained from FTIR spectroscopy. The immobilization of glucose oxidase (GOx) in particular was corroborated by analysis of the FTIR spectroscopy and C1s region in the XPS spectrum (Figures S1b and S2).

In the presence of glucose to the GelZyme, cascading electron transport from the enzyme sites to the electron‐conducting network is enabled (Figure 2d), either through direct electron transport (DET, enzyme sites‐conducting network) or indirect mediated electron transport (MET, enzyme sites‐ferrocene‐conducting network) [25]. This is achieved thanks to the significantly shortened 3D transport lengths in the GelZymes, compared to their 2D counterparts where enzyme membranes and electrodes are separated.

The above synthesis process delivers the GelZyme. For practical applications, their multifunctional properties must be characterized and validated. Below, these properties are categorized into electromechanical and electrochemical properties. The enzyme dynamics and strain robustness are also evaluated.

### 2.3 Electromechanical and Enzymatic Dynamics of GelZymes

**FIGURE 3:**
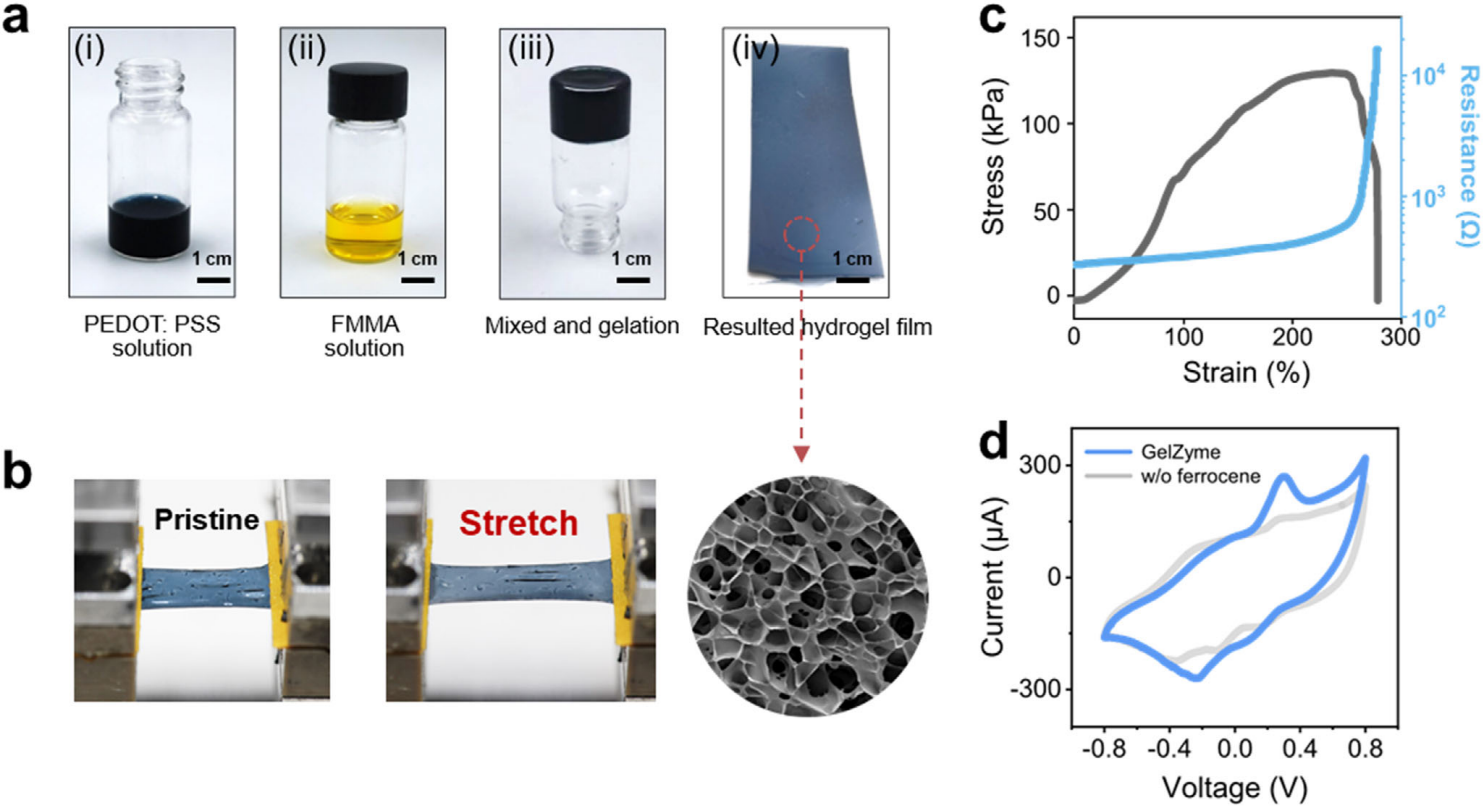
Electromechanical and electrochemical properties of GelZymes. a) Key steps in synthesizing GelZymes: (i) preparation of the PEDOT:PSS solution, (ii) addition of the redox mediator solution, and (iii and iv) fully assembled GelZymes with high porosity, promoting glucose molecule diffusion. b) Illustration of the stretchability of the GelZymes. c) Stress‐strain profiles of the GelZyme membranes, demonstrating their mechanical properties. d) Cyclic voltammetry curve of the GelZymes in the phosphate‐buffered saline (PBS) solution, with peaks indicating the successful grafting of ferrocene as a mediator to facilitate electron relay between enzymes and electrodes.

The GelZymes are elastoadhesive, as shown in Figure 2c. An electron conductivity of approximately ∼250 S/m was measured (Figure S3) which is maintainable up to 50% strain (Figure 3a–c) thanks to the constructed multi‐networked hydrogel structure. Moreover, the GelZyme shows a high porosity (Figure 3a), which is essential for biosensing applications as it facilitates the mass transport of target elements, such as glucose molecules.

To evaluate the overall electrochemical properties of the GelZyme, we measured its redox behaviors using cyclic voltammetry (Figure 3d). The results revealed that the GelZyme exhibited typical peaks of ferrocene species [26], which remained stable under up to 50% strain (Figure S4), demonstrating the robustness of the constructed 3D stretchable and conductive hydrogel electrodes. We also evaluated both the reproducibility and stability of the GelZyme thin film using cyclic voltammetry (CV). Reproducibility was confirmed across nine fabricated samples, which showed consistent electrochemical behavior (Figure S5). Additionally, the film exhibited excellent operational stability, maintaining its performance over 100 CV cycles with no significant loss of activity (Figure S6).

**FIGURE 4:**
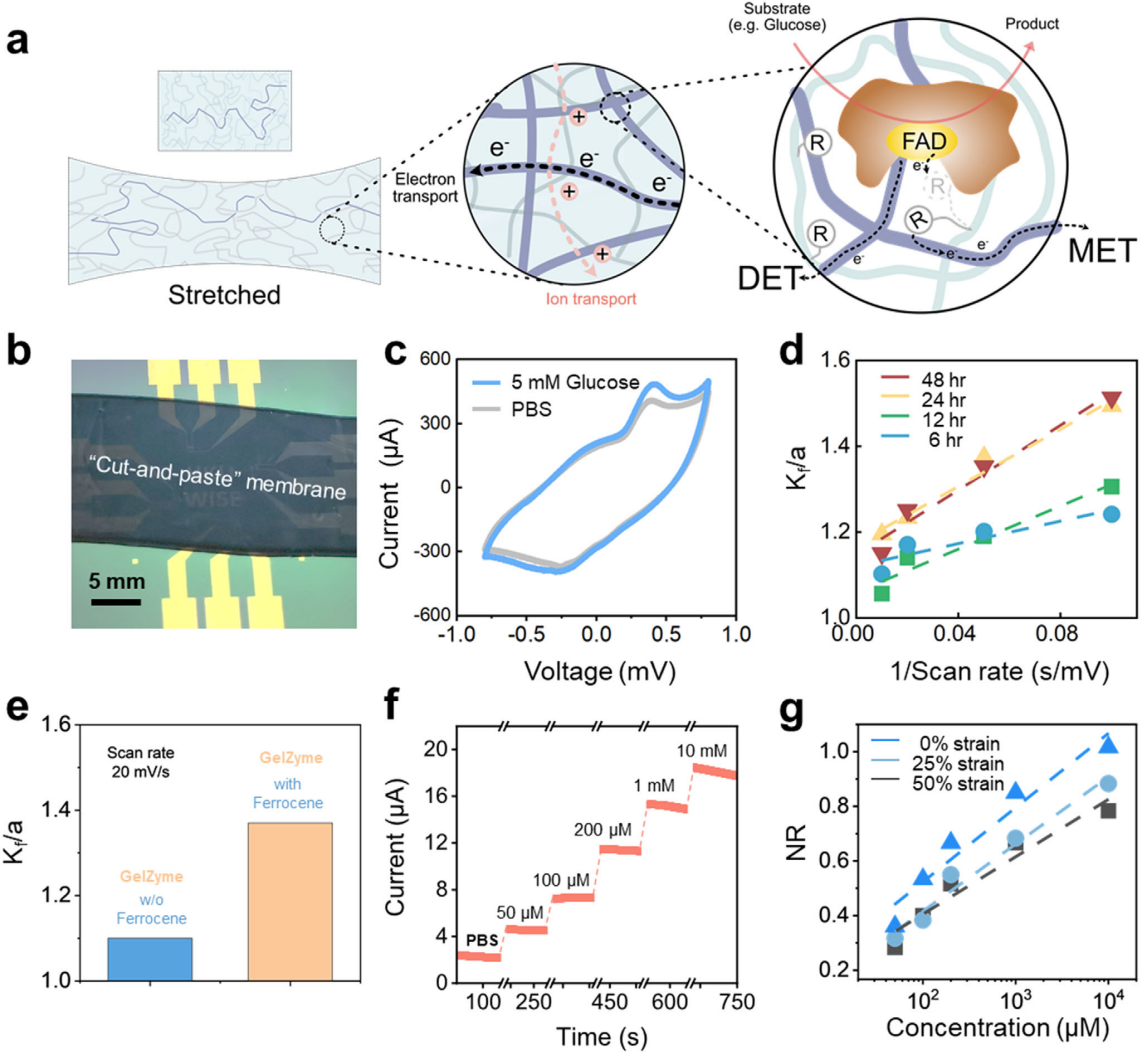
Enzymatic properties of GelZymes. a) Illustration of the electronic and ionic mixed‐conducting pathways in GelZymes and the enzymatic kinetics, including both direct electron transfer (DET) and mediated electron transfer (MET). b) Real image of the GelZymes, cut and applied to an array of sensing electrodes. c) The response of GelZymes to glucose (5 mM) in PBS solution. d) The kinetic parameters of GelZymes with different glucose oxidase enzyme loading times. Here, we use the experimental data, (i_cat_/i_d_)^2^, which is equal to K_f_/a, where a = nFv/RT. e) Comparison of the kinetic constant (K_f_) of GelZymes with and without ferrocene loading. The increase in K_f_ confirms the mediator role of ferrocene in GelZymes. f) The current flow through GelZymes was proportional to glucose concentration, demonstrating its capability for glucose sensing. g) Normalized current response (NR) of GelZymes in response to varying glucose concentrations and strain.

Significantly, a clear enzymatic catalytic current was observed in the presence of glucose [6] (Figure 4a–c), confirming the efficacy of GelZyme for enzymatic biosensing applications. Additionally, we evaluated the biocompatibility of the GelZyme using a live/dead cell staining assay (Figure S7).

A critical aspect to evaluate the enzymatic dynamics of GelZyme is to clarify the electrons transport mechanisms from the enzyme sites to the conducting network of the hydrogel electrodes. The transport process was promoted by the introduction of PEDOT:PSS conducting network to shorten the distance between the enzyme sites and the electrode (i.e., PEDOT^+^ conducting networks) [27]. This strategy can promote both the direct electron transfer (DET) and mediated electron transfer (MET) processes (illustrated in Figure 4a).

We first quantitatively investigated the kinetics of redox electron transports in the GelZyme by measuring the rate constants (K_*f*_) with the theory developed by Nicholson and Shain [28]. This model allows us to gain insight into the enzyme reactions on the basis of the following reaction scheme: $R-e^{-}↔O;Z+O\overset{k_{f}}{\to }R$; [6] where O and R refer to the respective redox forms of ferrocene, Z is the reduced enzyme. The K_*f*_can be calculated by comparing the ratio of the kinetic (*i*_cat_) to diffusion‐controlled current (*i*_d_):

(1) $$\frac{i_{\mathrm{cat}}}{i_{d}}=\frac{1}{0.447}\;{\left(\frac{RT}{nF}\right)}^{1/2}{\left(\frac{\sigma K_{f}c_{g}}{\nu \left[Z\right]}\right)}^{1/2}\;$$

where R is the universal gas constant, T is the temperature, n is the number of electrons, F is Faraday's constant, *c*_g_ is the substrate concentration (mM), σ is the stoichiometric coefficient and ν the potential sweep rate (V/s) [6, 28, 29].

The K_*f*_ of the GelZyme increases significantly with the increased loading of the enzyme, indicating the grafting of the enzymes onto the hydrogels (Figure 4d, at 0.10 s/mV). Meanwhile, a linear relationship between K_*f*_ and *v* ^−1^ was observed under different enzyme concentrations, consistent with Equation (1), confirming a complete 3D charge transport process within the GelZyme. The K_*f*_ fails to increase with prolonged enzyme loading times (48 h), indicating that enzyme‐loading process is saturated (Figure 4d).

Another important metric of the enzyme kinetics is the contributions from DET (from enzyme sites to PEDOT^+^ directly) and MET (from enzyme sites to ferrocene to PEDOT). To distinguish this, we compared the K_*f*_ of GelZymes, fabricated under the same conditions, but with and without ferrocene grafting (Figure 4e). The results show that introducing ferrocene mediators increases the K_*f*_. This increase is reasonable because the hydrogel, with its sparse polymer network—while essential for stretchability and molecular diffusion—is not inherently favorable for electron transport. In the presence of ferrocene, these mediators act as key electron relay sites through a collision process, thus enhancing electron transport efficiency.

We also examined the sensitivity of the GelZyme to glucose and its strain robustness. As shown in Figure 4f, the redox current of the glucose sensors, assembled with a 50 µm thick GelZyme membrane, showed good linearity with glucose concentrations ranging from 50 µM to 10 mM, which covers the glucose concentration range in various bodily fluids. Critically, the sensor's accuracy was evaluated using the Mean Absolute Relative Difference (MARD). The achieved MARD of approximately 6% (Figure S8) demonstrates high analytical accuracy. The GelZyme glucose sensor remains functional under cyclic strain conditions (0–50%) (Figure 4g), with a slight drop in sensitivity which can be attributed to strain‐induced permanent morphological changes of the hydrogel networks.

### 2.4 Scalable Fabrication of the GelZymes

To fully leverage the potential of GelZymes for non‐invasive, skin‐integrated biosensing applications, it is essential to enable scalable production using accessible and cost‐effective methods. In this context, we developed a one‐step, water‐processable fabrication protocol. First, the materials required for assembling the composite GelZyme are pre‐mixed in a single step. The GelZymes are then formed through a sequential process of photo‐crosslinking, solvent exchange, and enzyme loading processes. Pre‐setting the processing conditions during these steps allows us to fine‐tune the electromechanical and enzymatic properties of the hydrogels for a high yield.

**FIGURE 5:**
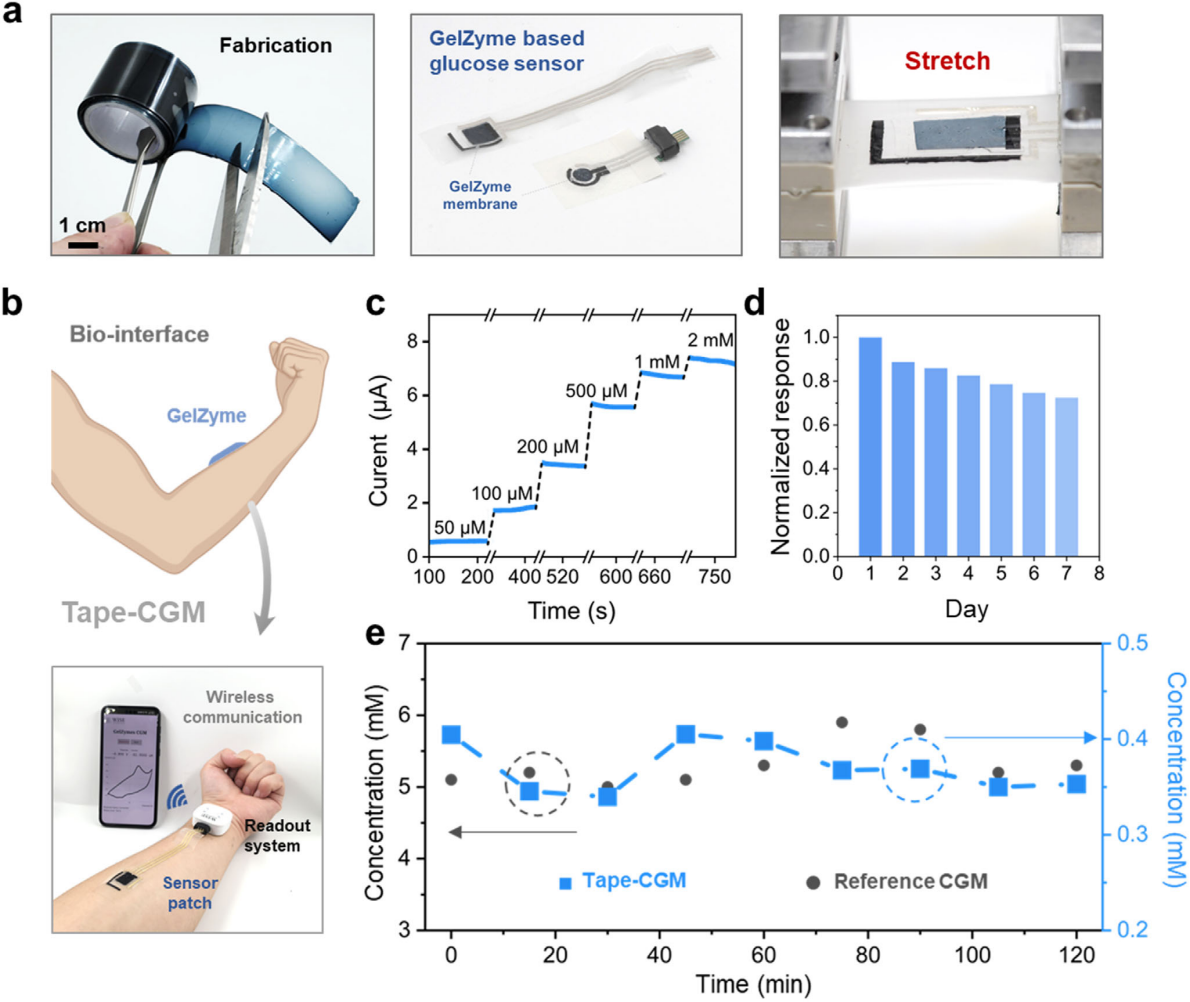
GelZymes enable the rapid fabrication of non‐invasive, tissue‐integratable glucose biosensors for wearable health applications. a) GelZymes can be fabricated into adhesive tapes, which can be easily “cut and pasted” onto pre‐fabricated electrodes to assemble glucose sensors. b) Schematic of a tape‐based continuous glucose monitor (CGM) using GelZymes and a wearable potentiostat. c) The GelZyme‐based tape CGM can detect glucose concentrations ranging from 50 µM to 2 mM. d) The stability performance of the tape‐CGM system over 7 days. e) Comparison of real‐time glucose monitoring results between the GelZyme‐based tape‐CGM system and a commercial Freestyle glucometer (in vivo). The slight mismatch in readings may be attributed to an impaired correlation between sweat glucose and blood glucose in the experimental setting or to uncontrolled sweat collection conditions.

With the above fabrication approach, we successfully mass‐produced GelZyme membranes (Figure 5a) with thicknesses controllable from 50 to 500 µm, and with arbitrary shapes (Figure S9). These GelZyme membranes can be easily shaped into different forms using a “cut‐and‐stick” approach, offering unique opportunities to simplify the assembly of enzyme biosensors. Their elastoadhesiveness, essential for mitigating motion artifacts, is evaluated in Figure S10. A 4‐fold increase in adhesion is observed compared to the reference gels after introducing tannic acid (TA) to provide dynamic hydrogen bonds, without affecting the enzymatic functions. All GelZyme membranes retain their glucose sensing functions under strain conditions, ensuring their suitability for practical tissue‐interfaced applications.

### 2.5 GelZyme Tapes for Quick Assembling of Wearable, Non‐Invasive CGMs

The successful fabrication of GelZyme, combined with the scalable production procedures described above, enables their immediate application in assembling non‐invasive skin‐integrated glucose biosensors (Figure 5; Figures S11 and S12). To fully leverage the unique properties of the GelZyme, including its stretchability and adhesiveness, we demonstrated a tape‐based continuous glucose monitor (tape‐CGM) by simply sticking the GelZyme on a medical tape (Figure S13). The tape‐CGM can thus be easily deployed on human skin as simply as applying as a medical tape (Figure 5b).

The tape‐based CGM (tape‐CGM) was seamlessly interfaced with a custom‐developed, wearable electrochemical readout unit (Figure S14) to enable continuous, real‐time data acquisition. The system's performance was demonstrated in a real‐time monitoring scenario (Figure S15). Furthermore, the mechanical robustness of the integrated GelZyme‐based CGM system was confirmed through stretchability tests, which verified its operational integrity under strain (Figure S16). Figure 5b,c shows that the tape‐CGM can detect glucose levels in human sweat (50 µM to 2 mM) under arbitrary motion conditions. The long‐term stability of the GelZyme‐based tape CGM sensor was evaluated over 7 days by monitoring its sensitivity to glucose concentrations ranging from 200 µm to 1 mm. As shown in Figure 5d, the system maintained stable performance throughout the testing period, with a small degradation in sensitivity compared to the first day. Then the results are benchmarked with reference data obtained from a commercial CGM for a period of 2 h, showcasing its potential for practical applications (Figure 5e). The slight mismatch in the reading can be attributed to an impaired correlation between sweat glucose and blood glucose in our experimental setting, or to uncontrolled sweat collection conditions.

## 3 Conclusion

In conclusion, we have presented GelZymes, which achieve two key breakthroughs compared to current enzyme electrodes: i) combining the enzyme membrane and electrode into a single 3D integrated electrode, thereby simplifying fabrication and eliminating interface stability issues; and ii) achieving tissue‐like viscoelastic properties—low modulus, stretchable and adhesive—which are rarely attained in existing rigid enzyme membranes.

The GelZyme is synthesized through a synthetic bioelectronic approach that integrates mixed‐conducting, multinetwork hydrogels with enzymes by: i) first, synthesizing a stretchable 3D hydrogel network; ii) second, introducing a secondary mixed‐conducting polymer network onto the hydrogel framework; and iii) third, loading enzymes and mediators onto the 3D hydrogel to relay the enzymatic process from the enzymes to the conducting network. Additionally, the adhesiveness of the GelZyme membranes was enhanced by introducing dynamic ionic species, such as TA.

We also developed a facile method for the scalable fabrication of GelZyme membranes for practical biosensing applications. These GelZyme membranes can be easily shaped into different forms using a “cut‐and‐stick” approach, offering unique opportunities to simplify the assembly of viscoelastic enzyme biosensors. Finally, we prototyped a tape‐CGM by simply attaching the GelZymes to medical tape for glucose monitoring in sweat or wounds.

Despite these advancements, several challenges remain before the broader application of GelZymes can be realized. For example, assembling GelZymes into functional devices requires integration with other functional layers, such as semipermeable membranes [30], which are often rigid and present challenges at the interfaces. Furthermore, it may be difficult to impart stretchability to these semipermeable membranes due to the critical requirements for controlled diffusion of the target analyte. The tradeoff between functionality and stretchability must be carefully addressed.

Nonetheless, we anticipate that the concept and synthetic strategy of GelZyme can pave the way for the development of similar gel‐based biosystems [31, 32] for a variety of non‐invasive, skin‐integratable biosensing applications.

## 4 Methods

### 4.1 Materials

PEDOT:PSS aqueous suspension (Clevios PH1000) was purchased from Heraeus. Acrylic acid (AA), acrylamide (AAM), N,N'‐Methylenebisacrylamide (MBAA), Poly(ethylene glycol) diglycidyl ether (PEGDE), ammonium persulphate (APS), glutaraldehyde (GA), Tannic acid (TA), β‐cyclodextrin (β‐CD), 2‐Hydroxy‐4′‐(2‐hydroxyethoxy)‐2‐methylpropiophenone (HMPP) were purchased from Sigma–Aldrich. Ferrocenylmethyl methacrylate (FMMA), sodium chloride (NaCl), Phosphate‐buffered saline (PBS), dimethylsulfoxide (DMSO), glucose oxidase (GOx), D‐(+)‐Glucose were purchased from Aladdin. Polydimethylsiloxane (PDMS, Sylgard 184) was purchased from Dow Corning. The semi‐permeable thermoplastic polyurethane (TPU) film was purchased from 3 m. The silver paste and carbon paste were purchased from Voltera Co. The Ecoflex 00–30 was purchased from Smooth‐On Inc.

### 4.2 Synthesis of GelZyme

The hydrogel precursor solution was prepared by homogeneously mixing AA/AAM, FMMA (1 wt.% relative to AA/AAM), MBAA (0.1 mol% relative to AA), PEDOT:PSS (6 wt.% relative to AA/AAM), and initiator APS in a glass vial using a Vortex Mixer. The homogenized precursor solution was then added in a custom‐made mold and subjected to UV irradiation for 10 min to form the hydrogel membrane. Subsequently, a DMSO solution (DMSO/DI water = 5:3 v/v) was added to the hydrogel membrane and allowed to stand for 5–10 min. The sample was then baked at 95°C for 15 h. After baking, the hydrogel membrane was immersed in DI water to remove the DMSO and rehydrate the membrane. The rehydrated hydrogel was incubated in PBS buffer containing 10 mg/mL GOx and 5 mg/mL PEGDE for 24 h at room temperature to immobilize the enzyme and form the GelZyme. Glucose sensing was performed using glucose solutions prepared in PBS buffer with varying concentrations ranging from 50 µM to 10 mM.

### 4.3 Fabrication of the Stretchable GelZyme‐Based Glucose Sensor

The electrodes for stretchable sensor patch fabrication were fabricated by sequentially screen‐printing various types of stretchable conductive inks onto a TPU film using a polyester screen with a mesh size of 200 and a mesh thickness of 125 µm. The detailed fabrication procedures are provided in Figure S9. Briefly, stretchable Ag paste was screen printed first as the conductive traces, followed by screen printing stretchable carbon paste as the working and counter electrodes. After that, Ag/AgCl paste was screen printed to form the reference electrode. The curing process was performed after each screen‐printing step under the following conditions: the Ag/AgCl paste was cured at 60°C for 30 min in a convection oven; the stretchable silver and carbon paste were cured at 60°C for 60 min. Finally, an Ecoflex layer was screen printed to define the electrode area. The final device was cured at 65°C for 10 min and then allowed to stabilize at room temperature before use. The GelZyme was then affixed to the surface of the working electrode and left to dry at room temperature for 6 h to ensure close contact.

### 4.4 Integration of the GelZyme‐Based Tape‐CGM

The microfluidic modules were fabricated using a laser cutter to pattern double‐sided medical adhesive tape (180 µm thick) and PET (50 µm thick) in a layer‐by‐layer assembly process (Figure S14). The GelZyme‐based sensor was attached to the microfluidic patch using the double‐sided medical adhesive tape.

The readout system was modified to meet the requirements of the tape‐based CGM system. The system was enhanced with customized firmware, incorporating functionalities for data sampling, filtering, and device control. The hardware was optimized by integrating an analog microcontroller unit (ADuCM355), a Bluetooth Low Energy (BLE) unit, and other essential components, all assembled onto a printed circuit board (PCB) using standard fabrication processes. The modified readout system was connected to the glucose sensor, via a flexible printed circuit board (FPCB) connector. The performance of the readout system was evaluated through in vitro experiments to monitor glucose concentrations.

### 4.5 Sweat Glucose Monitoring Using Wearable Tape‐CGM

For sweat monitoring using the tape‐CGM system, the stretchable glucose sensor was secured to the skin using the bio‐adhesive tape integrated into the microfluidic patch. After calibration of the glucose sensor, the system continuously monitored glucose levels for 2 h using the amperometry method. Following this, the tester consumed a meal, and 1 h later, the sweat glucose concentration was measured again.

To validate the performance of the tape‐CGM system, a comparison was conducted with a commercially available reference CGM system, the FreeStyle Libre. The FreeStyle Libre system uses a needle‐based sensor approximately 2.0 cm in length, which is inserted subcutaneously to monitor interstitial glucose levels. The sensor is integrated with an adhesive patch for secure skin placement, a supporting frame for structural stability, and a wireless transmitter for real‐time glucose data transmission to a paired device.

### 4.6 Electrochemical Method

Cyclic voltammetry (CV), and amperometry method (*i–t* mode) measurements were performed using CHI 660E and PalmSens systems. The working electrode consisted of a hydrogel sample coated onto an indium tin oxide (ITO) glass substrate. An Ag/AgCl electrode was employed as the reference electrode, while a platinum (Pt) plate was used as the counter electrode.

### 4.7 Scanning Electron Microscopy (SEM) Characterizations

The GelZyme samples were preconditioned by immersing them in deionized water to ensure sufficient swelling. The samples were frozen in liquid nitrogen and mechanically fractured to expose the cross‐section and longitudinal section for examination. Then, the samples were subjected to vacuum drying using a freeze‐drying process. The dried samples were then imaged using a Hitachi S3400N Variable Pressure Scanning Electron Microscope (VP‐SEM) operated at an accelerating voltage of 3 kV.

### 4.8 Mechanical Testing and Adhesion Testing

To evaluate the tensile performance of the GelZyme, it was shaped into a dumbbell form and tested at room temperature under a fixed strain rate. For interfacial toughness measurements, the as‐prepared GelZymes (100 mm in length, 25 mm in width) were adhered to the surfaces of porcine tissues. Double‐sided tapes, applied with cyanoacrylate glue, were used as stiff backing layers for both the adhered substrates and the GelZymes. Interfacial toughness was defined as the ratio of two times the plateau force (for a 180 degree peel test) or the plateau force (for a 90 degree peel test) to the width of the hydrogel samples.

### 4.9 Characterization

All testing samples were prepared on Si substrate. Both N1s and C1s spectra of GOx‐modified films were acquired using XPS. The sample was tested by the Thermo SCIENTIFIC Nexsa with a monochromatic Al Kα X‐ray source at 12 kV. Fourier‐transform infrared spectroscopy (FTIR) spectra were recorded using the Nicolet iS50 FTIR Spectrometer.

## Author Contributions

S.Z. conceived this project, acquired funding and supervised the whole research. B.C., S.D., D.L., X.Z, X.T., J.B., and I.P. designed and conducted the experiments and collected the data. B.C. synthesized the GelZymes. S.Z., B.C., and S.D. drafted the manuscript. All authors contributed to the revising of the manuscript.

## Conflicts of Interest

A US patent has been filed for this work (US Provisional Application No. 63/612,381).

## Supporting information




**Supporting File 1**: advs73641‐sup‐0001‐SuppMat.docx.

**Supporting File 2**: advs73641‐sup‐0002‐Supporting Video.mp4.

## Data Availability

All data supporting the findings of this study are available within the Article and its Supporting Information. Additional raw data generated in this study are available from the corresponding authors on reasonable request.
