# Supplementary material for: Improving the Skin‐Conformability of Wearable Continuous Glucose Monitors With Synthetic Hydrogel Electrodes
Source: Adv Sci (Weinh). 2026 Jan 22;13(16):e17501. doi: 10.1002/advs.202517501 (PMC13042557; doi:10.1002/advs.202517501)
Supplement: Supplementary file 1 — Supporting File 1: advs73641‐sup‐0001‐SuppMat.docx. [file ADVS-13-e17501-s001.docx]

Supplementary Information

**Improving the Skin-Conformability of Wearable Continuous Glucose Monitors with Synthetic Hydrogel Electrodes**

*Binbin Cui†, Shilei Dai**†, Ivo Pang, Dingyao Liu, Xinran Zhang, Jing Bai, Xinyu Tian, Shiming Zhang****

B. Cui, Dr. S. Dai, I. Pang, Dr. D. Liu, Dr. J. Bai, X. Zhang, Dr. X. Tian, Prof. S. Zhang

Department of Electrical and Electronic Engineering, The University of Hong Kong, Pokfulam, Hong Kong SAR, China

Prof. S. Zhang

State Key Laboratory of Pharmaceutical Biotechnology, The University of Hong Kong, Hong Kong SAR, China

†These authors contributed equally to this work.

*Corresponding author: (S. Zhang) Email: beszhang@hku.hk

**This PDF file includes:**

**Figure S1.** Fourier-transform infrared spectroscopy (FTIR) spectra of PEDOT:PSS thin film and GelZyme films.

**Figure S2.** The C1s XPS characterizations of the GelZyme samples.

**Figure S3.** Conductivity test of GelZymes with different PEDOT:PSS ratios.

**Figure S4.** CV curves of GelZyme under different strains.

**Figure S5.** Reproducibility of GelZyme thin film.

**Figure S6.** Electrochemical stability of GelZyme demonstrated over 100 CV cycles.

**Figure S7.**The biocompatibility of GelZymes was assessed by evaluating their cytotoxicity using a live/dead cell staining assay.

**Figure S8.** The MARD value of GelZymes-CGM.

**Figure S9.** Scalable fabrication method of GelZyme membrane.

**Figure S10.** The adhesion test of different GelZymes compositions.

**Figure S11.** Fabrication process of stretchable sensor patch.

**Figure S12.** Cyclic voltammetry curve of Stretchable glucose sensor.

**Figure S13.** The schematic diagram of the microfluidic structure.

**Figure S14.** The photograph of tape-CGM system and circuit diagram of readout system.

**Figure S15.** Assembly and form factor of the GelZyme-based CGM system

**Figure S16.** Operational stability of the integrated CGM system under mechanical strain.


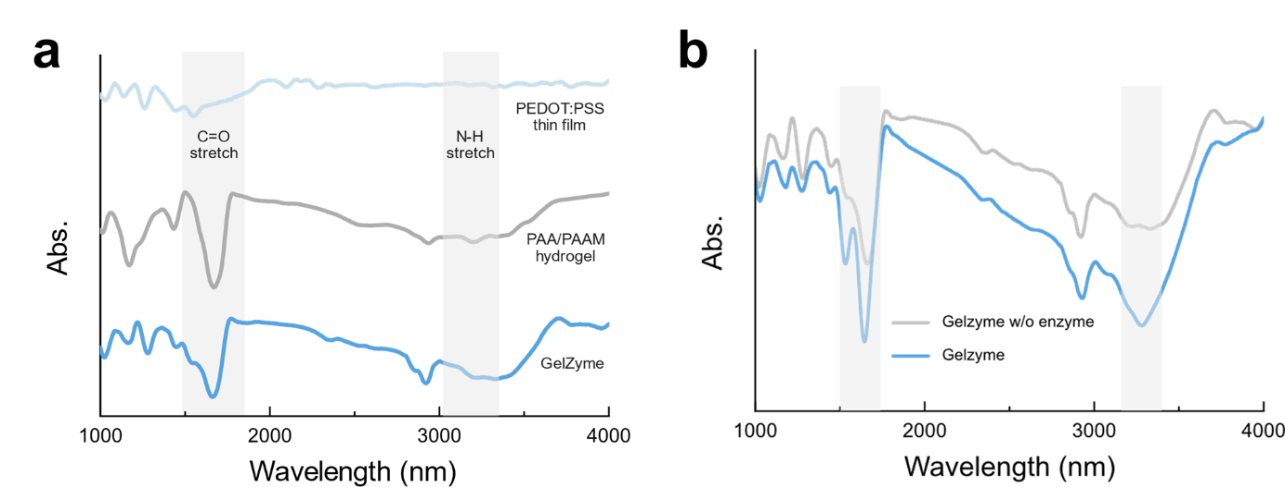


**Figure S1****. (a)** Fourier-transform infrared spectroscopy (FTIR) spectra of PEDOT:PSS thin film and GelZyme films. The successful incorporation of PAA/PAAM network is confirmed by the appearance of C=O and N-H stretch peak after UV crosslinking. **(b)** The FTIR spectra of GelZyme membrane with and without the enzyme immobilization.


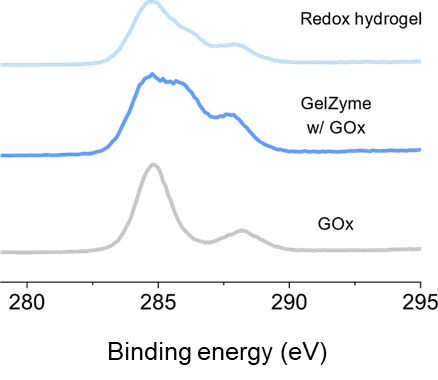


**Figure S2.** The C1s XPS characterizations on a redox hydrogel film (GelZyme without GOx immobilization), a GelZyme film (with GOx immobilization), and GOx alone.

**
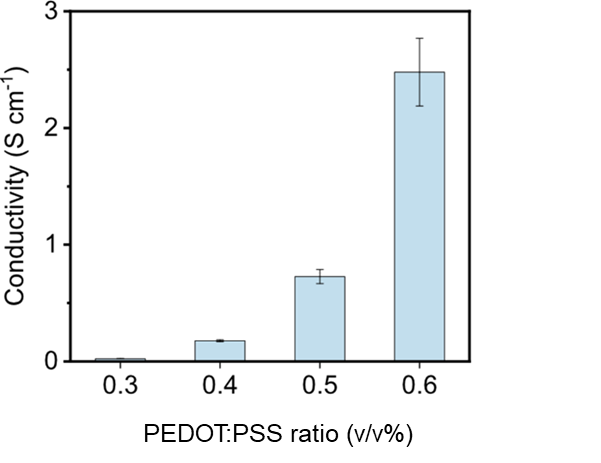
**

**Figure S3.** Conductivity test of GelZymes with different PEDOT:PSS ratios. The thickness of the GelZymes was about 5 μm in their dry state.


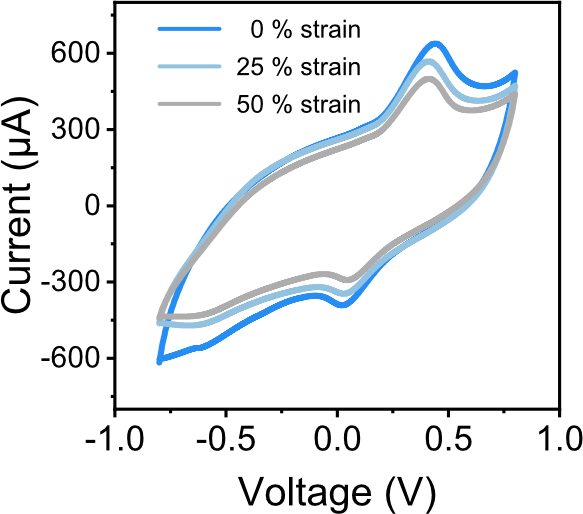


**Figure S4.** Cyclic voltammetry (CV) curves of GelZyme under different strains. The GelZyme exhibits a prominent redox peak at 0.4 V, at a scan rate of 20 mV/s.


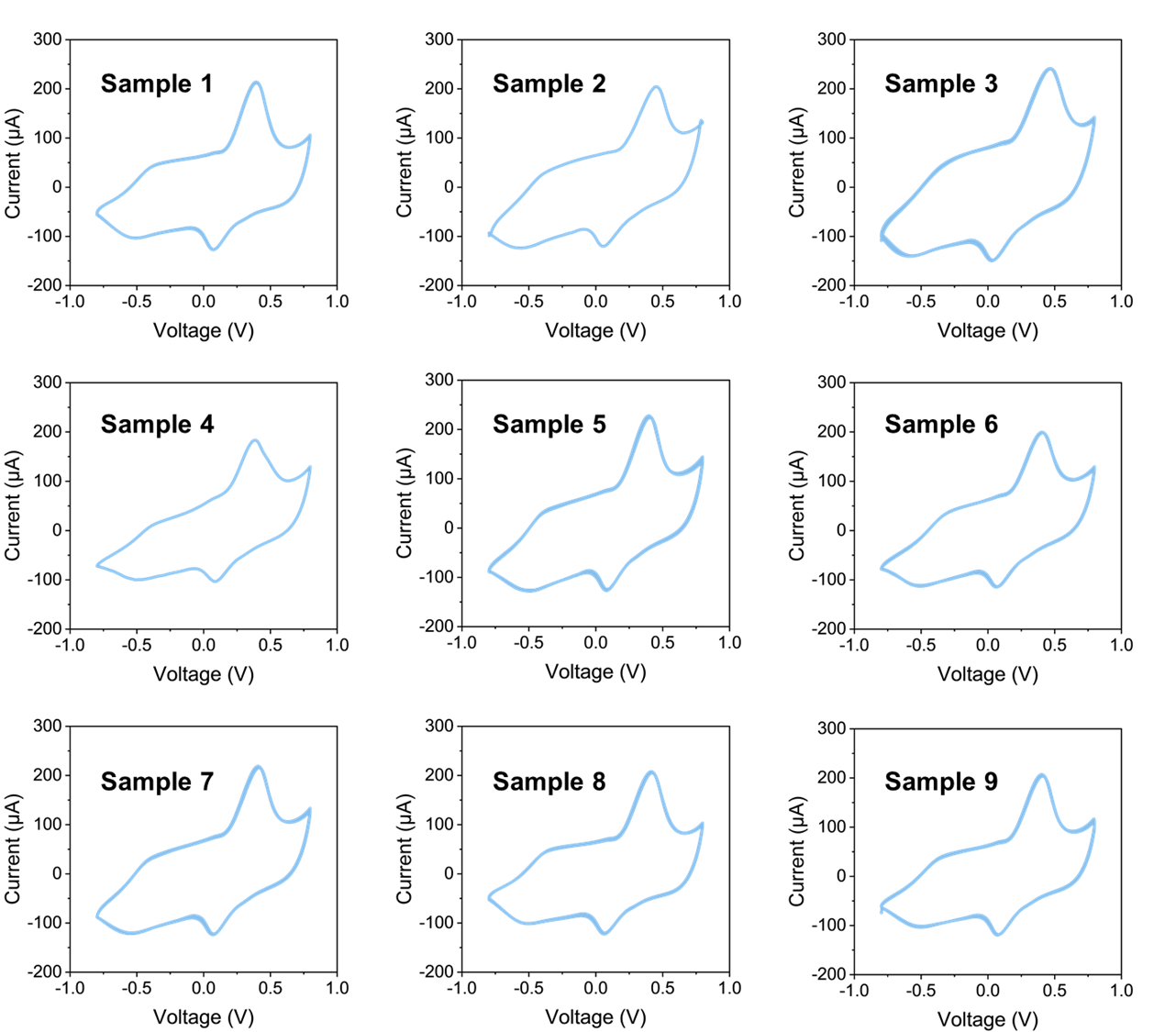


**Figure S5.** Reproducibility of GelZyme thin film, demonstrated by the similar CV curves of nine independent samples.


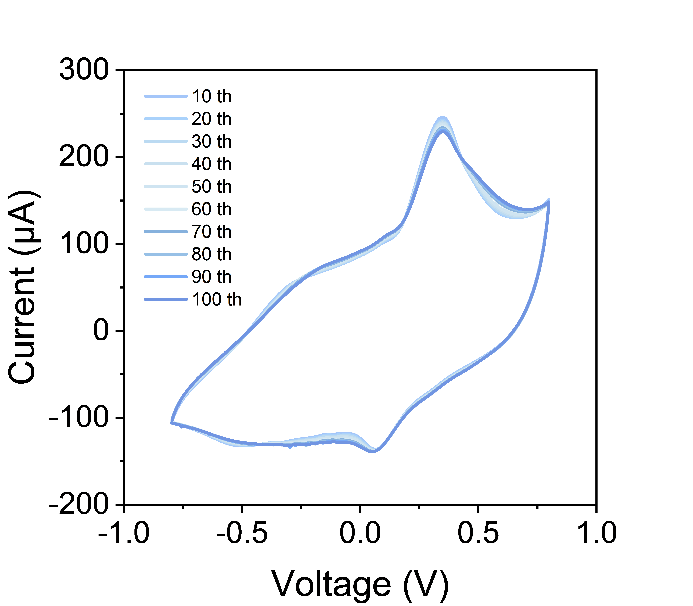


**Figure S6.** Electrochemical stability of GelZyme demonstrated over 100 CV cycles.

**
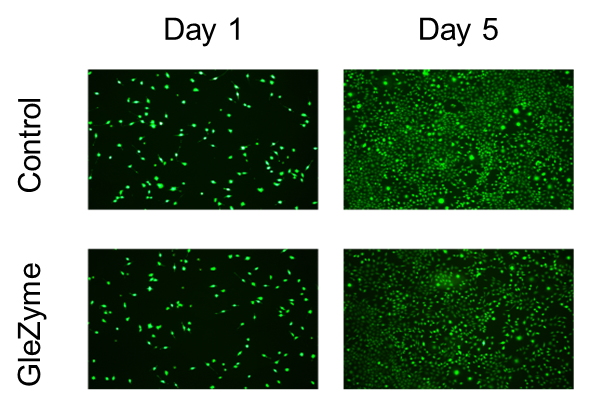
**

**Figure S7.** The biocompatibility of GelZymes, assessed by evaluating their cytotoxicity using a live/dead cell staining assay.


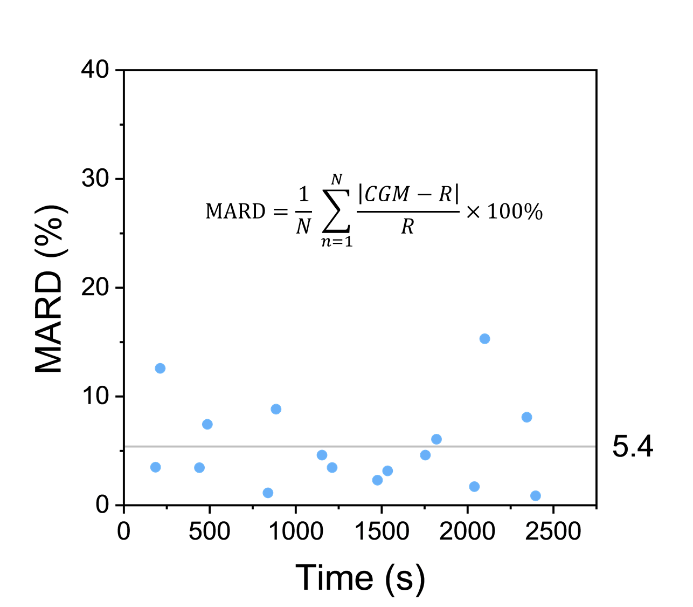


**Figure S8**. The MARD value of GelZymes-CGM (calculated with 16 sampling points within 2500s).

**
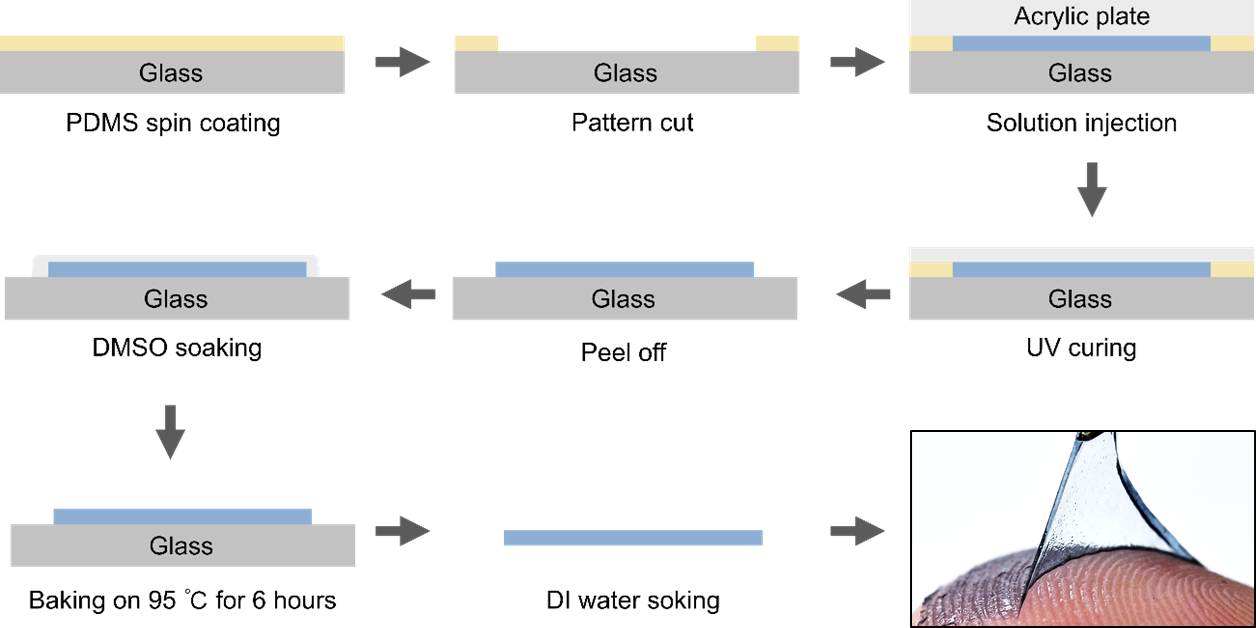
**

**Figure S9. Scalable fabrication method of GelZyme membrane.** First, drop-cast the PDMS solution onto a glass substrate and spin-coat it at different speeds to achieve varying PDMS thicknesses. Cut the PDMS into the desired shape to create wells for hydrogel fabrication. Add the hydrogel precursor solution into the wells and use an acrylic plate to confine the solution. Cure the hydrogel under UV light for 15 minutes, then peel off the acrylic plate and the PDMS film. Soak the hydrogel in a DMSO solution and bake it at 95°C for 6 hours. After baking, soak the hydrogel in deionized water to remove residual DMSO. The hydrogel is then ready for the enzyme solution soaking process.

**
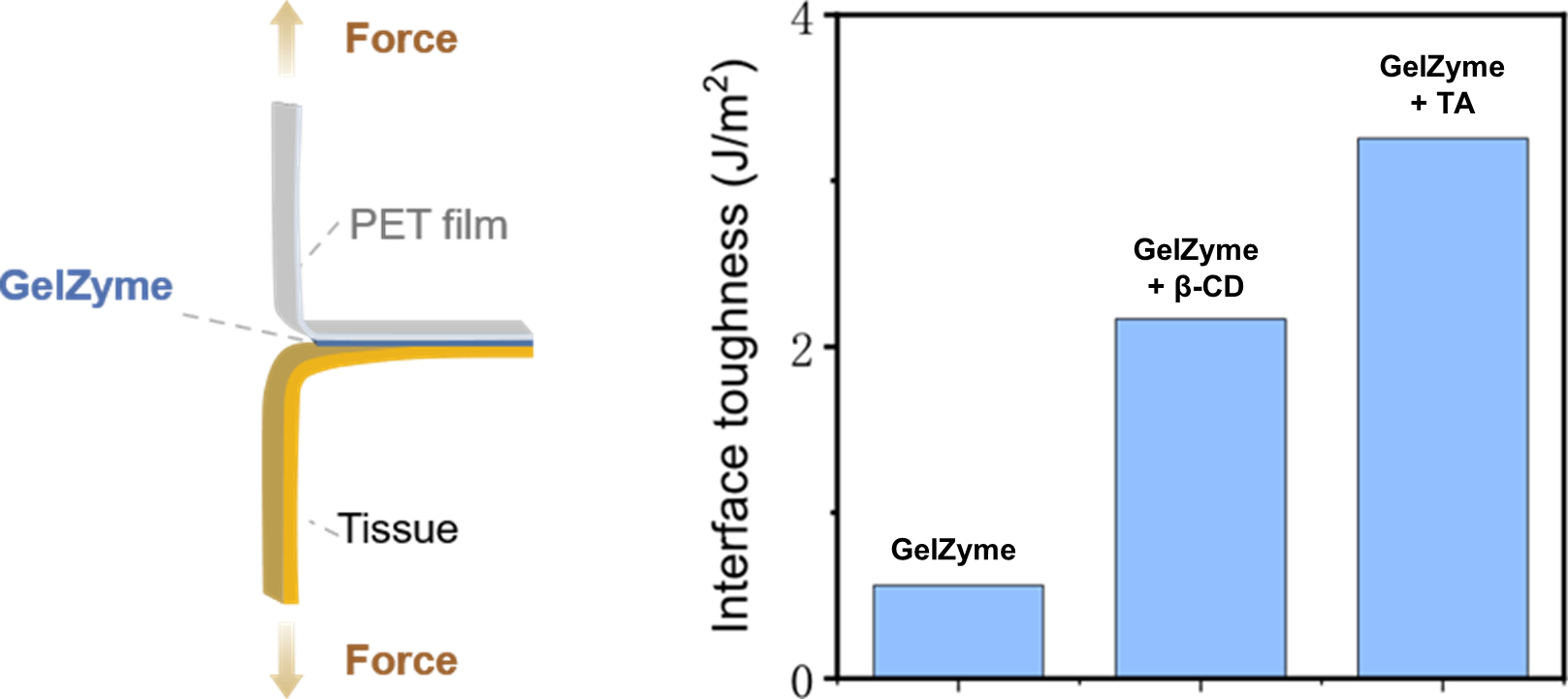
**

**Figure S10. The adhesion test of different GelZymes compositions.** The adhesion test of various GelZymes compositions demonstrated enhanced interface adhesion by incorporating specific additives. Tannic acid (TA) and β-cyclodextrin (β-CD), each at a 5% weight ratio relative to the AA/AAM content.


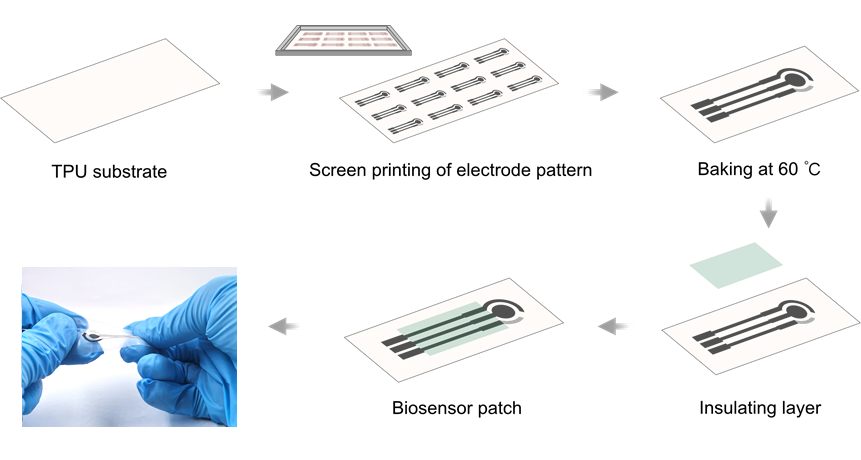


**Figure S11. Fabrication process of the stretchable sensor patch.** The fabrication process is described in the Methods section. The GelZymes will be applied to the working electrode for the sensing performance test.

**
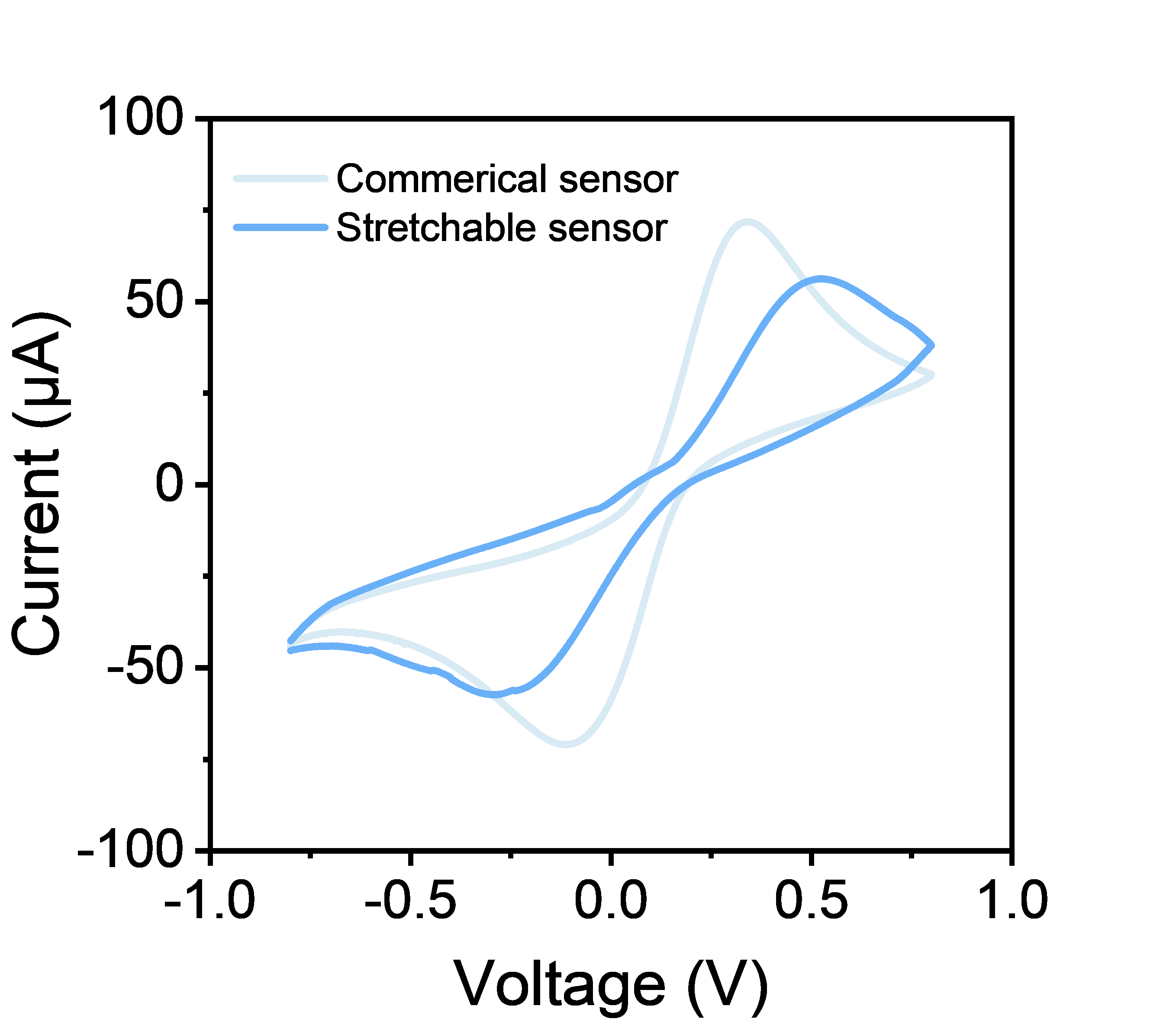
**

**Figure S12. Cyclic voltammetry (CV) curve of GelZyme-based stretchable glucose sensor.** The CV test was performed in a 5.0 mM K₃[Fe(CN)₆] solution. The scan rate was set and maintained at 20 mV/s throughout the experiment.

**
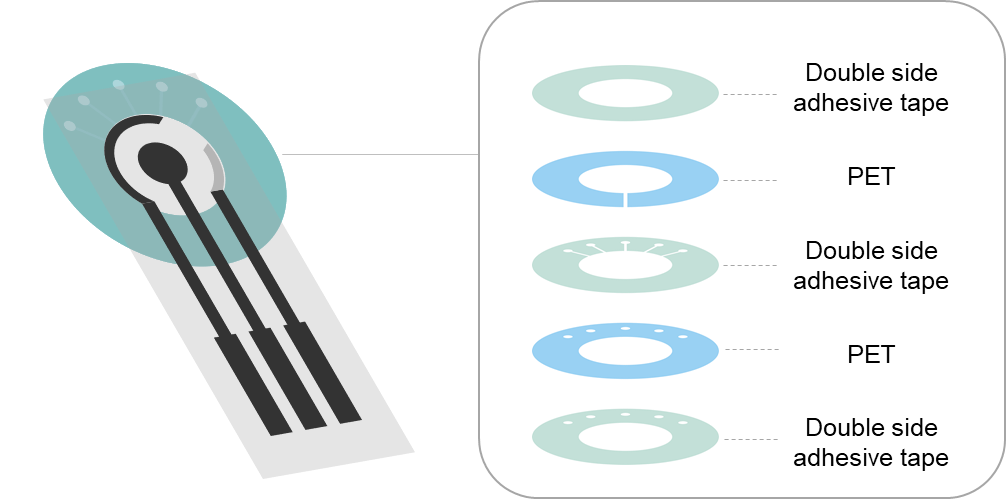
**

**Figure S13.** The schematic diagram of the microfluidic structure.

**
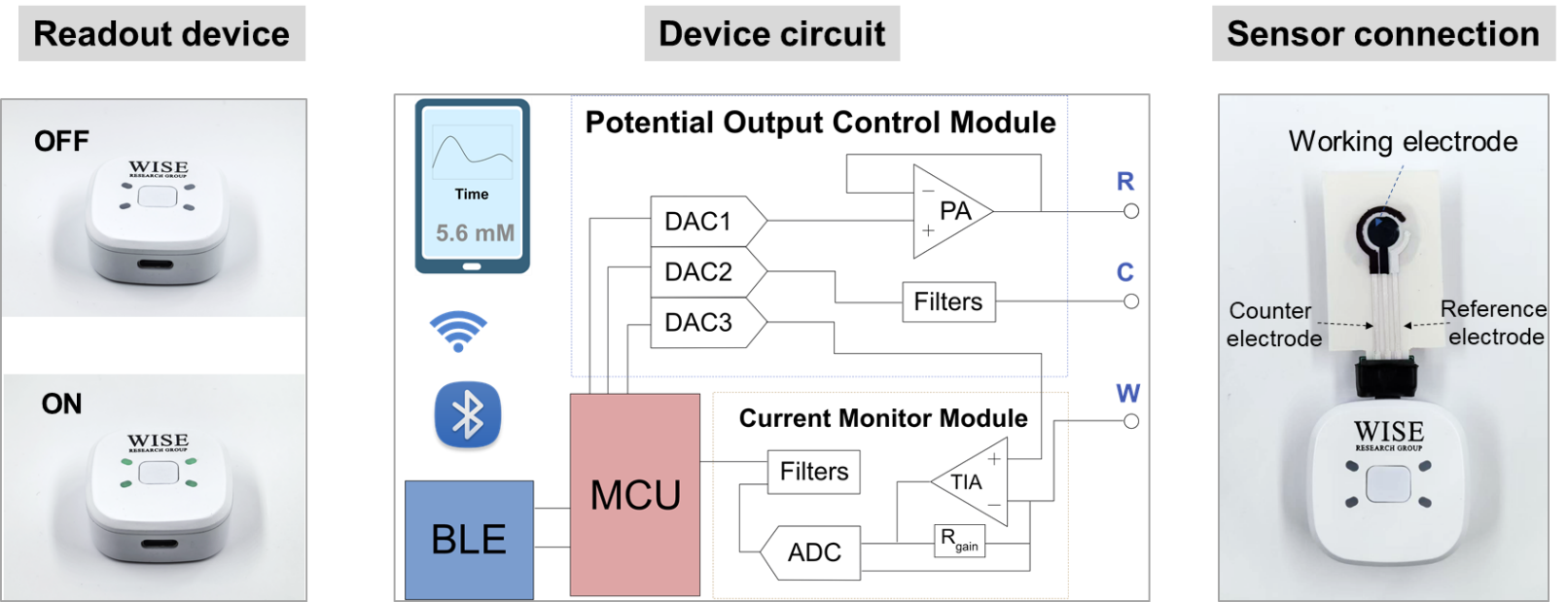
**

**Figure S14.** The photograph of the tape-CGM system and circuit diagram of the readout system.

**
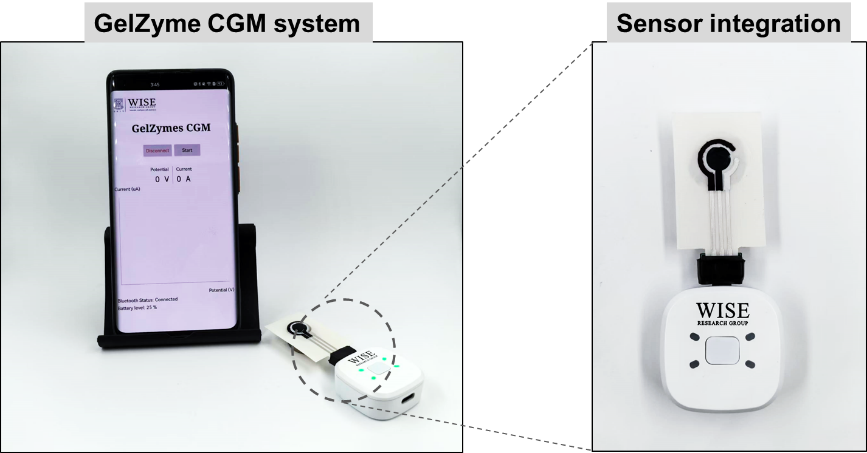
**

**Figure S15.** Assembly and form factor of the GelZyme-based CGM system, showing the sensor patch integrated with the wearable readout electronics.

**
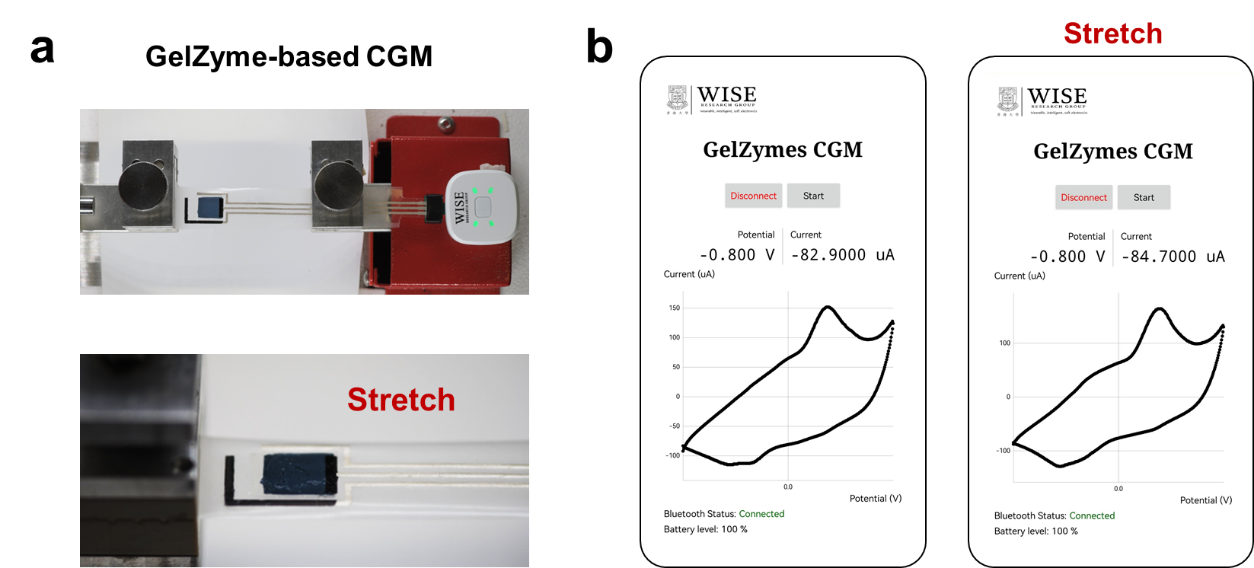
**

**Figure S16.** Operational stability of the integrated CGM system under mechanical strain. **(a)** The system maintains structural integrity under 20% strain. **(b)** Concurrently, the real-time cyclic voltammetry (CV) response remains stable, as displayed on the smartphone interface.
